# Two Rye Genes Responsible for Abnormal Development of Wheat–Rye Hybrids Are Linked in the Vicinity of an Evolutionary Translocation on Chromosome 6R

**DOI:** 10.3390/plants7030055

**Published:** 2018-07-10

**Authors:** Natalia V. Tsvetkova, Natalia D. Tikhenko, Bernd Hackauf, Anatoly V. Voylokov

**Affiliations:** 1 Department of Genetics and Biotechnology, St. Petersburg State University, Universiteskaya nab.7/9, St. Petersburg 199034, Russia; ntsvetkova@mail.ru; 2 Vavilov Institute of General Genetics Russian Academy of Sciences, St. Petersburg Branch, Universiteskaya nab.7/9, St. Petersburg 199034, Russia; tikhenko@mail.ru; 3 Leibnitz Institute of Plant Genetics and Crop Plant Research, Corrensstrasse 3, Stadt Seeland OT, D-06466 Gatersleben, Germany; 4 Julius Kühn-Institut, Institute for Breeding Research on Agricultural Crops, Rudolf-Schick-Platz 3a, D-18190 Sanitz, Germany; bernd.hackauf@julius-kuehn.de

**Keywords:** wheat-rye hybrids, genes of reproductive isolation, stem apical meristem, molecular marker

## Abstract

The post-zygotic reproductive isolation (RI) in plants is frequently based on the negative interaction of the parental genes involved in plant development. Of special interest is the study of such types of interactions in crop plants, because of the importance of distant hybridization in plant breeding. This study is devoted to map rye genes that are incompatible with wheat, determining the development of the shoot apical meristem in wheat–rye hybrids. Linkage analysis of microsatellite loci, as well as genes of embryo lethality (*Eml-R1*) and hybrid dwarfness (*Hdw-R1*) was carried out in hybrids of Chinese Spring wheat with recombinant inbred lines as well as interline rye hybrids. *Eml-R1* and *Hdw-R1* could be mapped proximal and distal of two closely linked EST-SSR markers, *Xgrm902* and *Xgrm959*, on rye chromosome 6R. Both rye genes are located on a segment of chromosome 6R that contains a breakpoint of evolutionary translocation between the ancestral chromosomes of homeologous groups 6 and 3. The obtained results are discussed in relation to genes interacting in developmental pathways as a class of causal genes of RI.

## 1. Introduction

Post-zygotic incompatibility in plants is often expressed in an autoimmune reaction (tissue necrosis) and disturbances of plant development, leading to a decrease in the viability or death of hybrids. Immunity and ontogenesis are controlled by many interacting genes. The interaction of genes is the basis of the canonical scheme (Bateson–Dobzhansky–Muller model), explaining the emergence and functioning of post-zygotic incompatibility. In cultivated and wild plants, a large number of genes controlling hybrid incompatibility in accordance with the classic two-locus scheme have been described [1]. The complementary interaction of incompatible alleles is established in interspecific and intraspecific hybrids. Phenotypes associated with hybrid necrosis resemble those elicited in response to various abiotic and biotic stresses [2]. However, while substantial progress has been achieved to uncover the molecular mechanisms by which disease resistance is achieved [2], the molecular mechanisms of hybrid incompatibility connected with the disturbances of development have not been studied in detail. The interaction of incompatible alleles of wheat (*Eml-A1*) and rye (*Eml-R1*) genes lead to ungerminating hybrid seeds. Mature seeds have normal endosperm, but hybrid embryos may be lacking, or are varying in size from small to normal, and are comprising of dead tissues, undifferentiated in the region of shoot apical meristem (SAM) [3]. Another rye mutation (*Hdw-R1b*) affects the shoot apical meristem in wheat–rye hybrids at the transition from vegetative to reproductive phases of development [4]. As a result, a phenotype develops similar to grass-clump dwarfs found in some intraspecific hybrids of bread wheat [5], and in hybrids of its progenitor species [6,7]. These wheat–rye hybrids stop development at the tillering stage, having three to five short tillers, and die within two months. Scanning electron microscopy showed that the apices of dwarf plants to that time do not reach the double-ridge stage, which is reminiscent of apices of young seedlings. The elucidation of the molecular control of developmental disorders in wheat–rye hybrids complements similar studies in related cereals, and should help unravel their causal function in the evolution of isolation mechanisms. The recently published draft genome sequence of rye [8] together with the high-quality reference genome sequence of barley [9] provide invaluable genomic resources to precisely characterize *Eml-R1* and *Hdw-R1* by their position in the rye genome. Here, we report on the identification of expressed sequence tag (EST) derived microsatellite markers, which are linked to both genes. Comparative mapping allowed to integrate the target interval in the barley genome sequence as a prerequisite for fine mapping and subsequent positional cloning.

## 2. Results

### 2.1. The Linkage of Mutant Genes with Molecular Markers

**Figure 1:**
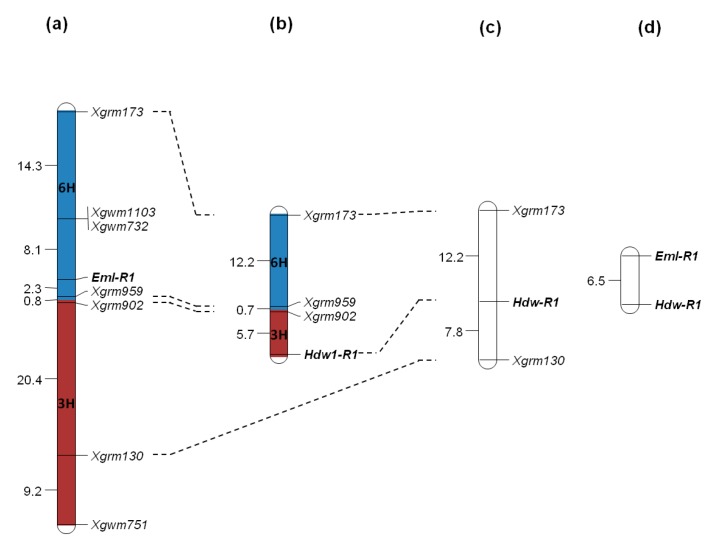
Genetic maps of *Eml-R1* and *Hdw-R1* on rye chromosome 6RL and their relationships with the homeologous barley chromosomes. Linkage maps were established for different wheat–rye hybrids: (**a**) CS × recombinant inbred lines (L2 × L7), *n* = 74; (**b**) CS × F1 (L6 × V1), *n* = 230; and (**c**,**d**) CS × F1 (V1 × L2), *n* = 230. Recombination frequency is shown in %.

The locus of hybrid embryo lethality, *Eml-R1*, has been localized previously on chromosome 6R [10,11] based on the linkage to the co-segregating markers *Xgwm1103/Xgwm732*. In the present study, we have mapped *Eml-R1* distal of *Xgwm1103/Xgwm732*, and closely linked to *Xgrm959* and *Xgrm902* in hybrids of Chinese Spring (CS) wheat, and a set of rye recombinant inbred lines (RILs) originating from the cross between L2 × L7 (Figure 1). We were able to integrate three additional microsatellite markers—*Xgrm173*, *Xgrm130*, and *Xgwm751*—in this linkage group on chromosome 6R. The loci *Xgrm173* and *Xgrm959–Xgrm902* carry different alleles in lines L6 and V1. This enabled testing the linkage of *Hdw-R1* with these markers in a CS × F1 (L6 × V1) cross. The markers revealed linkage to *Hdw-R1*. This gene, compared with *Eml-R1*, is located distal of the linked markers *Xgrm959–Xgrm902* (Figure 1b). Thus, both genes of hybrid incompatibility may be inherited together. Their joint segregation was validated in three hybrid populations.

### 2.2. Comparative Mapping

Each of the four EST-derived SSR markers *Xgrm173*, *Xgrm959*, *Xgrm902*, and *Xgrm130* could be integrated in the draft of the rye genome sequence (Table 1). The length of the corresponding Lo7 contigs varies between 362–11,532 bp; two of these contigs have been mapped on chromosome 6R. Sequence similarity searches in the barley genome sequence revealed that the orthologs of *Xgrm173* and *Xgrm959*, both flanking *Eml-R1*, are residing on chromosome 6H, while the *Hdw-R1* flanking markers *Xgrm902* and *Xgrm130* correspond to segments on chromosome 3H (Table 1, Figure 1).

### 2.3. Joint Segregation Analysis of Hybrid Dwarfness and Embryo Lethality

For segregation analysis, the germinating seeds of three populations with dihybrid segregations CS × F1 (V1 × L2), CS × F1 (L2 × V1), and CS × F1 (L2 × V10) were divided into five phenotypic classes (Table 1).

The ratio of seeds with normal embryos to seeds with abnormal embryos (with undifferentiated embryos and without embryos) corresponds to the expected monohybrid segregation in all of the studied hybrids. The segregation for the gene of hybrid dwarfness [4] can be observed only in plants grown from the seeds with normal alive embryos. In theory, the ratio of dwarf to normal plants depends on the linkage of genes *Eml-R1* and *Hdw-R1*. If these genes are not linked (segregate independently), we will observe monohybrid gametic segregation 1 (*Hdw-R1a*):1 (*Hdw-R1b*). In reality, we observed a case that was attributed to the tight linkage of the studied genes, which follows from their map position (Figure 1a,b). A large fraction of dwarf plants and a significantly smaller fraction of normal plants were found in the progeny of each cross (Table 2). Gametes, producing the normal wheat–rye hybrids, appear as a result of crossing over between these linked genes in the meiosis of the rye parent. With this assumption, we calculated a recombination frequency between the rye genes *Eml-R1* and *Hdw-R1* as a frequency of normal hybrid plants (rye recombinant gametes) among all of the hybrid plants (the all rye gametes). This frequency is equal to 6.5 ± 1.7% (Figure 1d), 3.2 ± 1.6%, and 9.0 ± 2.6% in the studied dihybrid cross combinations CS × F1 (V1 × L2), CS × F1 (L2 × V1), and CS × F1 (L2 × V10), respectively. As a mean value, one may consider 6.3 ± 1.1%, which was calculated for the pooled sample. A recombination rate between genes *Xgwm173* and *Hdw-R1* calculated in hybrids with monohybrid and dihybrid segregations do not differ to a large extent, and are equal to 17.9% and 12.2%, correspondingly. It is important to note that the frequency of seeds with normal differentiated embryos, but that are not capable of germination, varied significantly between these hybrids: 6.6%, 15.5%, and 69.5%. The appearance of this phenotypic class in segregation and its variable frequency are attributable to environmental variation. The fertilization and embryo development under distant hybridization are very sensitive to variability in temperature, humidity, and mineral supply. For this reason, the development of embryos carrying the normal *Eml-R1a* allele may be disturbed before seed maturity, and such embryos die. The mutant *Eml-R1b* allele expresses far earlier, and its expression leads to the development of morphologically distinct embryos that also die before maturity.

## 3. Discussion

In an initial attempt, we have mapped *Eml-R1* on chromosome 6RL based on linkage to the genomic wheat microsatellite markers *Xgwm1103/Xgwm732* [10,11]. In the present study, we describe for the first time that *Eml-R1* is linked to another gene controlling post-zygotic reproductive isolation between wheat and rye, *Hdw-R1*. Furthermore, the integration of EST-derived rye SSR marker enabled comparative mapping, and revealed that both genes are located on an interstitial region on chromosome 6RL, covering a previously reported 3L/6L translocation breakpoint [12,13]. While *Eml-R1* is residing on a segment that is homeologous with barley chromosome 6H, *Hdw-R1* maps distally from *Eml-R1* on a 6RL segment that is homeologous with the long arm of 3H. The 3L/6L translocation breakpoint is located within a 0.8-cM interval defined by *Xgrm959* and *Xgrm902*, respectively. The localization of both genes on different ancestral segments shaping the modern chromosome 6R is further supported by the genomic wheat microsatellite loci *Xgwm751*, *Xgwm1103,* and *Xgwm732*. The co-segregating microsatellite markers *Xgwm1103*/*Xgwm732* in wheat are located on chromosomes 6A [14] and 6D [15]. In contrast, marker *Xgwm751* was mapped in wheat close to the centromere on chromosome arm 3AL, and on the long arm of chromosome 3B [16]. The progress achieved in the present study concerning the localization of *Eml-R1* and *Hdw-R1* with respect to the position of translocation breakpoints is important in terms of understanding the mechanisms of reproductive isolation and the evolution of the rye karyotype. In *Saccharomyces cerevisiae*, chromosomal rearrangements have been identified as a major mechanism to reproductively isolate different strains [17]. In plants, knowledge of chromosomal rearrangements is still scarce, and their importance for speciation is controversial discussed [18]. Recently, 4L/5L translocation breakpoints have been comprehensively described at the molecular level as two hotspots of chromosomal rearrangements that have been reused during Triticeae evolution [19]. *Eml-R1* and *Hdw-R1* reside at the 3L/6L translocation in rye, and highlight that the fitness of wheat/rye hybrids can be genetically affected via embryogenesis or the vegetative development, respectively. As a consequence, both genes might have contributed to the speciation of rye, which diverged from *Triticum aestivum* approximately three to four million years ago [20]. The molecular genetic control of hybrid inviability in plants is yet not well understood [21]. The natural genetic diversity of rye inbred lines in the *Eml-R1* as well as *Hdw-R1* genes and a sophisticated phenotyping system based on test-crosses with wheat enables a forward genetics approach to isolate genes involved in the reproductive isolation of Triticeae species. With the recent availability of a draft genome sequence of rye [8] and a high-quality reference genome sequence of barley [9], the positional cloning of both genes has now become a feasible task in the large and complex rye genome. 

There are numerous examples of hybrid incompatibility manifesting itself as an arrest of plant development at different stages [1]. They include embryo and seedling lethality, failure to transition from vegetative to reproductive stages of development, or forming reproductive organs. Some of the described examples closely resemble the expression of known mutant genes controlling plant development through SAM maintenance and function. The death of hybrid plants at different stages of development was frequently connected with the necrosis of tissues, suggesting an autoimmune reaction. Thus, it is not easy to find the true cause of hybrid incompatibility. One key to solve the problem is an approach based on the identification of candidate genes. That approach allows the unraveling of complex hierarchical relationships of genes performing different functions, using the knowledge of the functions of the interacting candidate genes. Thus, the identification of the corresponding genes is critical for understanding the molecular mechanisms of complementary negative interactions in hybrids. Transcriptional analysis of the incompatibility of hybrids of tetraploid wheat and wild diploid relatives illustrates well the need for identification of the causal genes of incompatibility [6,7,22,23]. The authors describe the changes in the transcription activity of the hundreds of genes at shoot apices in hybrids between tetraploid wheat (*Triticum turgidum* ssp. *durum*, AABB genome) variety Langdon, and two wheat diploid relatives, *Aegilops tauschii* (DD genome) and *Aegilops umbellata* (UU genome). F1 hybrids of wheat with some accessions of both wild relatives show one of two developmental abnormalities: severe growth abortion (SGA), which may be considered as seedling lethality, and grass-clump dwarfness/hybrid necrosis. Both have some features closely resembling the morphological expression of the wheat–rye dwarfness. Lethal at a three-leaf stage and temperature independent SGA connected with the down-regulation of numerous transcription factors, including the KNOTTED1-like homeobox gene, maintaining SAM, and the cell cycle-related genes functioning in SAM and leaf primordia. The temperature-dependent grass-clump dwarfness is explained by the down-regulation of the APETALA-like MADS box genes, known as flowering promoters, and by increased miR156 transcription, leading to a reduced level of target mRNA of *SPL* genes (Squamosa promoter binding protein-box transcription factors), some of which promote tillering. It is very interesting that the grass-clump dwarf phenotype is characteristic of hybrids, growing under normal temperature conditions. The same hybrids at low temperature express a typical autoimmune response connecting with the repression of cell division. Transcription factors, such as small RNA, are capable of physically interacting with target DNA and RNA, correspondingly, and their genes can be considered as the most likely for the role of candidate genes. It is worth noting that the cited authors revealed the presence of compatible and non-compatible genotypes in all of the parents, but carried out the segregation analysis and mapped only one of the genes (*Net2*) in *Aegilops tauschii*. We found only incompatible alleles in Chinese Spring bread wheat and both types of alleles in the rye inbred lines. The wheat gene *Eml-A1*, which is complementary to rye incompatible allele *Eml-R1b* in the expression of hybrid embryo lethality, was mapped on the distal part of the long arm of chromosome 6A with the aid of the deletion lines of CS [24]. One would expect that *Eml* genes may have orthologs on the chromosomes of homeologous group 6 in different species of the tribe Triticeae, including one with sequenced genomes. The comprehensive study of our, and the cited, examples of genome incompatibility would resolve the subject under discussion. Namely, would the genes of developmental pathways be considered as a separate class of plant genes that is capable of serving as causal genes for reproductive isolation? The progress in this direction is closely connected with genome sequencing in species of tribe Triticeae. Rye now does not limit the comparative studies, owing to new genomic resources [8].

## 4. Materials and Methods

### 4.1. Plant Material

For segregation analysis, the wheat–rye hybrid seeds were produced in two types of crosses. The female parent in both cases was bread wheat of the Chinese Spring variety, as the male parents were interline rye F1 hybrids, or the set of 74 L2 × L7 RILs were used. It was shown previously that rye lines L6 and L7 carry normal (compatible) alleles in both studied genes (*Eml-R1a* and *Hdw-R1a*). Line L2 carries the incompatible allele *Eml-R1b*, which leads to embryo lethality in hybrids with Chinese Spring wheat. Lines V1 and V10 carry the incompatible allele of hybrid dwarfness *Hdw-R1b*. To produce wheat–rye hybrid seeds, wheat spikes were emasculated 1–2 days before anthesis, and pollinated 2–4 days later with freshly collected rye pollen. Wheat plants were pollinated by pollen collected from individual plants of corresponding F1 hybrids, or each of 74 RIL plants.

### 4.2. Phenotyping and Genotyping

Mature wheat–rye caryopses were soaked in water, and 3–4 days later, the embryos were classified as normal (completely differentiated) or abnormal (undifferentiated or without embryos). To study the segregation for dwarfness, the seeds with normal alive embryos were sown in the soil, and one month old plants were differentiated as either dwarf or normal. DNA was isolated from the leaves of grown plants using the CTAB method [25]. For each hybrid combination, the polymorphic microsatellite markers were selected on the basis of preliminary screening, and data for the linkage of *Eml-R1* with two co-segregating markers *Xgwm1103/Xgwm732* on chromosome 6R [8,9]. In all, three wheat microsatellites (*Xgwm1103/Xgwm732*, and *Xgwm751*) and four rye ones (*Xgrm173*, *Xgrm959*, *Xgrm902*, and *Xgrm130*) were used for mapping. Segregation for each marker corresponded to the expected gametic ratio of 1:1 (*p* > 0.05) in hybrids of wheat with RILs and with rye F1 L6 × V1. The segregation for the markers *Xgrm173* and *Xgrm130*, which were studied in hybrids CS × (V1 × L2), differed to a large extent from the monohybrid ratio. In this hybrid, markers may be studied only in segregating dwarf plants and rare recombinant plants with normal phenotypes. Information for the used wheat microsatellite (GWM) belongs to the Institute of Plant Genetics and Crop Plant Research (Gatersleben, Germany). A set of EST-derived rye microsatellites (GRM) were used according to Martis et al. [12]. Electrophoresis was performed in 6% denaturing polyacrylamide gel on an automatic laser fluorescent sequencer ALFexpress II (Amersham-Pharmacia-Biotech, Amersham, UK). The sizes of the fragments were calculated using the program Fragment Analyser 1.02 (Amersham-Pharmacia-Biotech) by comparison with internal standards of known size.

### 4.3. Linkage Map Construction and Comparative Mapping

Segregation for the studied genes and markers were tested by χ^2^. Linkage groups for different hybrids were built by MultiPoint3.3 (MultiQTL Ltd., Institute of Evolution, Haifa, Israel, http://www.multiqtl.com). A recombination frequency in percent was used as a measure of the genetic distance. For comparison purposes, RF per single meiosis was calculated for RILs. To identify the orthologous *Eml-R1* and *Hdw-R1* segments in the genomes of barley and rye, rye EST assemblies representing the GRM markers [12] were compared against masked barley pseudomolecules [9] as well as rye whole genome shotgun contigs v2 [8] using BLASTN and the IPK barley and rye blast server (http://webblast.ipk-gatersleben.de/).

## Figures and Tables

**Table 1:** Integration of the 6R markers derived from rye EST sequences in the rye and barley genome sequences.

Marker ^a^	BLASTN Query ^a^	Rye				Barley			
		Subject	Expect	Chr ^b^	pos	Subject	s_start	s_end	Expect
GRM0173	Sce_Assembly02_c6346	Lo7_v2_contig_257767	1 × 10^−100^	0R		chr6H	570137757	570137532	6 × 10^−59^
GRM0959	Sce_Assembly02_c87163	Lo7_v2_contig_4801	3 × 10^−145^	6R	108.7	chr6H	579212782	579213063	9 × 10^−43^
GRM0902	Sce_Assembly02_c81266	Lo7_v2_contig_1427427	1 × 10^−104^	0R		chr3H	19821332	19821201	2 × 10^−12^
GRM0130	Sce_Assembly02_c4514	Lo7_v2_contig_126444	5 × 10^−167^	6R	124.6	chr3H	674285409	674285721	5 × 10^−122^

^a^ According to Martis et al., 2013; ^b^ according to Bauer et al., 2017.

**Table 2:** Segregation for hybrid dwarfness (*Hdw-R1*) and embryo lethality (*Eml-R1*) in crosses of Chinese Spring wheat with F1 interline rye hybrids.

Hybrid Combination	Seeds with Normal Embryo (*Eml-R1a*)			Seeds with Abnormal Embryo (*Eml-R1b*)		χ^2^ 1 *Eml-R1a*: 1 *Eml-R1b*
	Alive		Dead	Undifferentiated Embryo	Without Embryo	
	Dwarf	Normal				
CS × F1(V1 × L2)	215	15	34 (6.6) *	244	10	1.19
CS × F1(L2 × V1)	121	4	51 (15.5) *	145	7	1.76
CS × F1(L2 × V10)	111	11	228 (65.9) *	321	37	0.09

* Percentage is shown in brackets.
